# Multiprincipal Element M_2_FeC (M = Ti,V,Nb,Ta,Zr) MAX Phases with Synergistic Effect of Dielectric and Magnetic Loss

**DOI:** 10.1002/advs.202206877

**Published:** 2023-02-02

**Authors:** Lu Chen, Youbing Li, Biao Zhao, Shanshan Liu, Huibin Zhang, Ke Chen, Mian Li, Shiyu Du, Faxian Xiu, Renchao Che, Zhifang Chai, Qing Huang

**Affiliations:** ^1^ Engineering Laboratory of Advanced Energy Materials Ningbo Institute of Materials Technology and Engineering Chinese Academy of Sciences Ningbo Zhejiang 315201 China; ^2^ College of Materials Science and Opto‐electronic Technology University of Chinese Academy of Sciences 19 A Yuquan Rd, Shijingshan District Beijing 100049 China; ^3^ Qianwan Institute of CNiTECH Ningbo 315336 China; ^4^ Laboratory of Advanced Materials Shanghai Key Lab of Molecular Catalysis and Innovative Materials School of Microelectronics Fudan University Shanghai 200438 China; ^5^ State Key Laboratory of Surface Physics and Department of Physics Fudan University Shanghai 200433 China

**Keywords:** dielectric loss, electromagnetic wave absorption, magnetic loss, magnetic MAX phases, multiprincipal elements

## Abstract

Electromagnetic (EM) wave pollution is harmful to human health and environment, thus it is absolutely important to develop new electromagnetic wave absorbing materials. MAX phases have been attracted more attention as a potential candidate for electromagnetic wave absorbing materials due to their high conductivity and nanolaminated structure. Herein, two new magnetic MAX phases with multiprincipal elements ((Ti_1/3_Nb_1/3_Ta_1/3_)_2_FeC and (Ti_0.2_V_0.2_Nb_0.2_Ta_0.2_Zr_0.2_)_2_FeC) in which Fe atoms replace Al atoms in the A sites are successfully synthesized by an isomorphous replacement reaction of multiprincipal (Ti_1/3_Nb_1/3_Ta_1/3_)_2_AlC and (Ti_0.2_V_0.2_Nb_0.2_Ta_0.2_Zr_0.2_)_2_AlC MAX phases with Lewis acid salt (FeCl_2_). (Ti_1/3_Nb_1/3_Ta_1/3_)_2_FeC and (Ti_0.2_V_0.2_Nb_0.2_Ta_0.2_Zr_0.2_)_2_FeC exhibit ferromagnetic behavior, and the Curie temperature (*T*_c_) are 302 and 235 K, respectively. The dual electromagnetic absorption mechanisms that include dielectric and magnetic loss, which is realized in these multiprincipal MAX phases. The minimum reflection loss (RL) of (Ti_1/3_Nb_1/3_Ta_1/3_)_2_FeC is −44.4 dB at 6.56 GHz with 3 mm thickness, and the effective bandwidth is 2.48 GHz. Additionally, the electromagnetic wave absorption properties of the magnetic MAX phases indicate that magnetic loss also plays an important role besides dielectric loss. This work shows a promising composition‐design strategy to develop MAX phases with good EM wave absorption performance via simultaneously regulating dielectric and magnetic loss together.

## Graphical abstract

Substitution of Fe for Al in multiprincipal MAX phases exhibits room‐temperature ferromagnetic and excellent electromagnetic absorption behavior due to the contribution of both dielectric and magnetic losses.

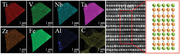

## 1 Introduction

With the widely use of wireless communication and electronic equipment, the problem of electromagnetic (EM) wave pollution is becoming increasingly serious, which has great harm to human health and the environment.^[^ ^1^ ^]^ It is necessary to design and fabricate effective EM wave absorbers in order to solve these problems. M*_n+1_* AX*_n_* (MAX) phases, a family of nanolaminated ternary‐layered transition metal carbides/nitrides with hexagonal crystal structure (space group P6_3_ *mm/c*, No. 194), where M is an early transition metal, A is mainly from groups 13–16, X is C and/or N, and *n* = 1, 2, or 3.^[^ ^2^ ^]^ Generally, the unique crystal structure and the mixed covalent and ionic M‐X bond in MAX phases contribute to their high electrical/thermal conductivities and ceramic‐like structural properties.^[^ ^3^ ^]^ Due to the peculiar crystal structure, MAX phases possess easy machinability,^[^ ^4^ ^]^ good electrical and thermal conductivity,^[^ ^5^ ^]^ high‐temperature oxidation resistance, and stability. MAX phases have been considered as a potential EM wave absorbing materials and are attracting great attention of researchers in the past decades.^[^ ^4^, ^5^ ^]^

Up to now, a few MAX phases, such as Ti_3_SiC_2_,^[^ ^4^ ^]^ Ti_3_AlC_2_,^[^ ^5^ ^]^ Cr_2_AlC,^[^ ^6^ ^]^ and their compounds,^[^ ^7^ ^]^ have been studied, and showed their great potential to be excellent EM microwave absorbers. In order to improve the EM wave absorption performance, a series of MAX phases have been designed and synthesized through tuning in chemical compositions and structures.^[^ ^8^ ^]^ In 2021, Kong's et al.^[^ ^9^ ^]^ found that (V_1‐x‐y_Ti_x_Cr_y_)_2_AlC MAX phase exhibited prominent EM absorption performance when compared with ternary MAX phases (Ti_2_AlC, V_2_AlC, and Cr_2_AlC). Moreover, high entropy (Mo_0.25_Cr_0.25_Ti_0.25_V_0.25_)_3_AlC_2_ MAX phase kept excellent EM wave absorption even after high‐temperature oxidation treatment.^[^ ^10^ ^]^ Those above work indicated that the EM microwave absorption performance of MAX phases can be adjusted by tuning the composition elements of transition metals. In addition to dielectric loss, magnetic loss is also a way to enhance the electromagnetic attenuation capability of electromagnetic absorbers.^[^ ^11^ ^]^ However, to our knowledge, MAX phases, as a typical dielectric loss absorber, are ignored the magnetic loss on the role of electromagnetic wave absorption. Preparing new MAX phases that combine dielectric and magnetic loss mechanisms is still a great challenge. Therefore, it is imperatively to develop magnetic MAX phases that can attenuate EM wave via both dielectric and magnetic loss mechanism.

Herein, multiprincipal (Ti_1/3_Nb_1/3_Ta_1/3_)_2_FeC and (Ti_0.2_V_0.2_Nb_0.2_Ta_0.2_Zr_0.2_)_2_FeC MAX phases were first topotactically transformed into magnetic MAX phases of (Ti_1/3_Nb_1/3_Ta_1/3_)_2_FeC and (Ti_0.2_V_0.2_Nb_0.2_Ta_0.2_Zr_0.2_)_2_FeC via isomorphous replacement reaction of precursors with Lewis acid salt (FeCl_2_). These magnetic MAX phases exhibit unusual ferromagnetic behavior and high Curie temperature. Moreover, (Ti_1/3_Nb_1/3_Ta_1/3_)_2_FeC shows excellent EM wave absorption with a minimum reflection loss (RL) value of −44.4 dB at 6.56 GHz (thickness is 3 mm), and the effective bandwidth is 2.48 GHz.

## 2 Results and Discussion

### 2.1 Preparation and Characterization of Magnetic MAX Phases with Multiprincipal Elements

**Figure 1:**
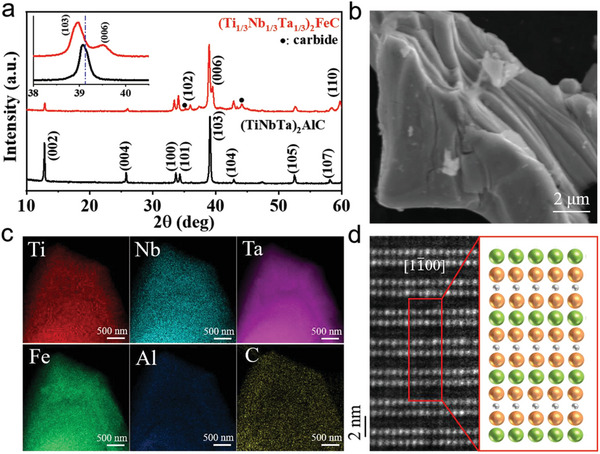
Phase compositions and microstructure of (Ti_1/3_Nb_1/3_Ta_1/3_)_2_AlC and (Ti_1/3_Nb_1/3_Ta_1/3_)_2_FeC MAX phases. a) XRD patterns of (Ti_1/3_Nb_1/3_Ta_1/3_)_2_AlC and (Ti_1/3_Nb_1/3_Ta_1/3_)_2_FeC, respectively. And insert is partial enlarged perspective of XRD patterns. b) SEM image of (Ti_1/3_Nb_1/3_Ta_1/3_)_2_FeC. c) EDS mapping in TEM model of Ti‐*K*_*α*_, Nb‐*L*_*α*_, Ta‐*L*_*α*_, and Fe‐*K*_*α*_, respectively. d) Aberration corrected (HR)‐STEM imaging and atomic lattice‐resolved EDS analyzing of (Ti_1/3_Nb_1/3_Ta_1/3_)_2_FeC in [1$\bar{1}$00] direction. and the yellow, green, and gray balls represent atoms in the M‐, A‐, and X‐sites, respectively.

First, (Ti_1/3_Nb_1/3_Ta_1/3_)_2_AlC MAX phases with multiprincipal elements were prepared by the molten salt method (Figure S1, Supporting Information). **Figure** **1**a shows the X‐ray diffraction (XRD) patterns of (Ti_1/3_Nb_1/3_Ta_1/3_)_2_AlC MAX phase before and after reaction with FeCl_2_ Lewis acid salt. Compared with (Ti_1/3_Nb_1/3_Ta_1/3_)_2_AlC, substitution of Fe atoms for Al atoms in final product makes no change in crystal structure what is of evidence in almost identical X‐ray diffraction pattern. However, for (TiNbTa)_2_FeC, e.g., the (100) and (101) diffraction peaks, are slightly shifted toward lower angles, showing that the expanded lattice parameters after the replacement reaction of Al atoms by Fe atoms. In addition, the diminishing peaks of (002) and (004) planes but appearance of (006) plane indicates lowered short‐distance ordering but comparable long‐distance ordering along the *c* axis in the Fe‐substituted MAX phase. The refined lattice parameters of (TiNbTa)_2_FeC were *a* = 0.3094 nm and *c* = 1.3682 nm, respectively (reliability factors are *R*_wp_ = 13.56%, *R*_p_ = 10.18%), which are a little larger than that of (Ti_1/3_Nb_1/3_Ta_1/3_)_2_AlC (*a* = 0.3078 nm and *c* = 1.3843 nm), were shown in Figures S2–S3 and Tables S1–S2 (Supporting Information), respectively. The scanning electron microscopy (SEM) image of (TiNbTa)_2_FeC exhibits typical nanolaminated structure of traditional MAX phases (Figure 1b). And corresponding energy‐dispersive spectrometer (EDS) results indicate that the atomic portions of Ti, Nb, Ta, Fe, and C were 8.0, 6.3, 7.5, 18.5, and 45.7 at%, respectively (Figure S4 and Table S3, Supporting Information). Subsequently, the content of Al is 0.3 at% and the atomic ratio of ((Ti + Nb + Ta):Fe) is close to 2:1, which is close to stoichiometry of the (TiNbTa)_2_AlC precursor, indicating the full‐replacement of Al by Fe in the final product. The refined molar ratio (Table S4, Supporting Information) of M‐site solid solutes in (TiNbTa)_2_FeC phase was 0.332:0.334:0.334, which could be considered the chemical formula can be written as (Ti_1/3_Nb_1/3_Ta_1/3_)_2_FeC.

The atomic structure and chemical analysis of (Ti_1/3_Nb_1/3_Ta_1/3_)_2_FeC was further identified through a high‐resolution‐scanning transmission electron microscope (HR‐STEM) together with lattice‐resolved EDS. The element mapping in TEM‐EDS mode confirms that all the transition metal elements of MAX phases are uniformly distributed without Al element (Figure 1c). Along the vertical direction of both images, it can be observed that single layers of atomic columns (the A layers) are interleaved by two adjacent layers of bright atomic columns (the M layers). As the brightness of the atom is dependent on its mass (intensity ≈ Z^2^), carbon is typically not visible because of its weak electron‐scattering nature as compared with the heavier M and A atoms. Since the brightness of the atom is directly related to its atom mass, the Fe atoms are hardly distinguished between the (TiNbTa)_2_C slabs. Thus, lattice‐resolved EDS line profile (Figure S5, Supporting Information) verifies that the A sites are occupied by Fe atoms and the formation of magnetic (TiNbTa)_2_C MAX phase (Figure 1d), which is consistent with XRD results.

**Figure 2:**
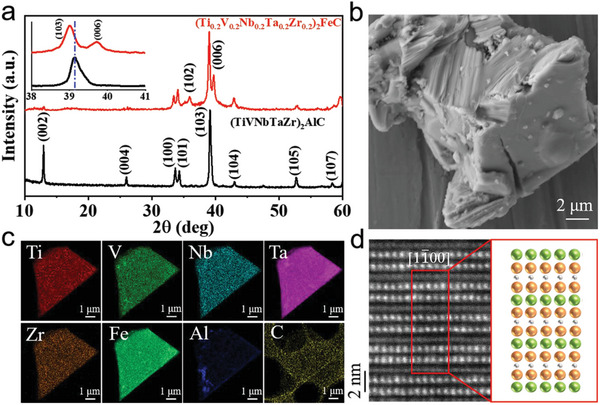
Phase identification and microstructure of (Ti_0.2_V_0.2_Nb_0.2_Ta_0.2_Zr_0.2_)_2_FeC magnetic MAX phases. a) XRD patterns of (Ti_0.2_V_0.2_Nb_0.2_Ta_0.2_Zr_0.2_)_2_AlC and (Ti_0.2_V_0.2_Nb_0.2_Ta_0.2_Zr_0.2_)_2_FeC, respectively. And insert is partial enlarged perspective of XRD patterns. b) SEM image of (Ti_0.2_V_0.2_Nb_0.2_Ta_0.2_Zr_0.2_)_2_FeC. c) EDS mapping in TEM model of Ti‐*K*_*α*_, V‐*K*_*α*_, Nb‐*L*_*α*_, Ta‐*L*_*α*_, Zr‐*L*_*α*_, and Fe‐*K*_*α*_, respectively. d) Aberration‐corrected (HR)‐STEM imaging and atomic lattice‐resolved EDS analyzing of (Ti_0.2_V_0.2_Nb_0.2_Ta_0.2_Zr_0.2_)_2_FeC in [1$\bar{1}$00] direction. And the yellow, green, and gray balls represent atoms in the M‐, A‐, and X‐sites, respectively.

The rich chemical composition of MAX phases provides the opportunity for flexible choice of M elements (e.g., Ti, Nb, Ta, Zr, and Hf). Herein, multiprincipal (Ti_0.2_V_0.2_Nb_0.2_Ta_0.2_Zr_0.2_)_2_AlC MAX phase precursor was also synthesized, as shown in Figure S6 (Supporting Information). Then, (TiVNbTaZr)_2_FeC was synthesized following the same isomorphous replacement reaction. **Figure** **2**a shows XRD patterns before and after the reaction of (Ti_0.2_V_0.2_Nb_0.2_Ta_0.2_Zr_0.2_)_2_AlC with FeCl_2_ molten salt, which exhibits a similar behavior of (Ti_1/3_Nb_1/3_Ta_1/3_)_2_FeC with the relative peak intensity of the (000*l*) changed significantly. And the Rietveld refinement (Figures S7–S8 and Tables S5–S6, Supporting Information) of XRD patterns of the (Ti_0.2_V_0.2_Nb_0.2_Ta_0.2_Zr_0.2_)_2_AlC and (TiVNbTaZr)_2_FeC MAX phases yields a reliability factor *R*_wp_ of 14.50% and 10.51%, respectively. Lattice parameters are *a* = 0.3095 nm and *c* = 1.3640 nm for (TiVNbTaZr)_2_FeC. The SEM image of (TiVNbTaZr)_2_FeC (Figure 2b) is agreement with (Ti_0.2_V_0.2_Nb_0.2_Ta_0.2_Zr_0.2_)_2_AlC. Following the results of the refinement and EDS spectrum (Table S7, Supporting Information), the chemical formula was confirmed as (Ti_0.2_V_0.2_Nb_0.2_Ta_0.2_Zr_0.2_)_2_FeC. Furthermore, the EDS results (Figure 2c and Table S8, Supporting Information) indicated that the disappearance of Al signals, and the atomic ratio of ((Ti+V+Nb+Ta+Zr):Fe) is close to 2:1. In addition, the STEM images and related STEM‐EDS analysis (Figure 2d; Figures S9 and S10, Supporting Information) are further evidence of the successful synthesis of (Ti_0.2_V_0.2_Nb_0.2_Ta_0.2_Zr_0.2_)_2_FeC.

### 2.2 Magnetic Properties of Magnetic MAX Phases with Multiprincipal Elements

**Figure 3:**
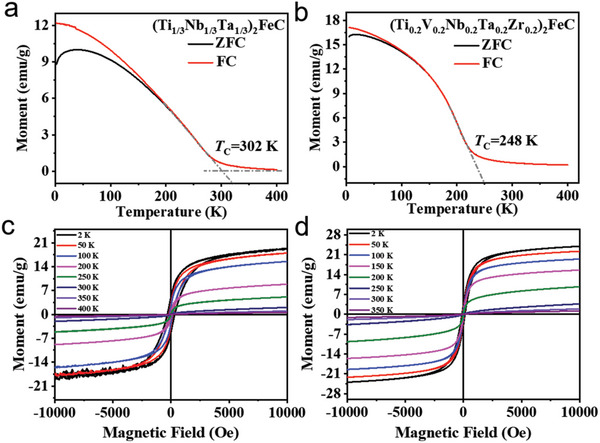
Magnetic behavior of new magnetic MAX phases. Zero‐field‐cooling curves (black lines) and field cooled curves (red lines) of (Ti_1/3_Nb_1/3_Ta_1/3_)_2_FeC and (Ti_0.2_V_0.2_Nb_0.2_Ta_0.2_Zr_0.2_)_2_FeC are illustrated in (a) and (b). c,d) Shows field‐dependent magnetization curves of above the two MAX phases at different temperature from −10 000 to 10 000 Oe.

For the purpose of understanding the change in intrinsic magnetic properties of these magnetic MAX phases, temperature‐dependent magnetization (*M*(*T*)) curves (**Figure** **3**a,b) were collected at 1000 Oe in the range of 2–400 K, where zero‐field‐cooling (ZFC) curves were marked in black, and field‐cooling (FC) curves were marked in red. Notably, bifurcation during the low‐temperature region between the FC and ZFC magnetization curves can be easily observed, and two magnetization curves gradually overlapped and became indistinguishable as temperature increases. The disordered in the ZFC process at low temperature is mainly caused by external magnetic field. The bifurcation and irreversible behavior further indicated the presence of spin glass transition behavior in the magnetic MAX phases.^[^ ^12^ ^]^

Magnetization fells down gradually with increasing temperature, which revealed that magnetic properties of MAX phases were from ferromagnetic to paramagnetic with increasing temperature. In addition, Curie temperature (*T*_c_) was defined as the temperature point correlated with transformation changes of *M*(*T*) curves.^[^ ^13^ ^]^ And the *T*_c_ of (Ti_0.2_V_0.2_Nb_0.2_Ta_0.2_Zr_0.2_)_2_FeC was obtained by extrapolation with values of 235 K, while the *T*_c_ of (Ti_1/3_Nb_1/3_Ta_1/3_)_2_FeC is 302 K. The powder of (Ti_1/3_Nb_1/3_Ta_1/3_)_2_FeC can hang on the wall of glass bottle hangs on the wall when it is close to the magnet (Figure S11, Supporting Information). Compared with reported magnetic‐ternary MAX phases^[^ ^8^, ^14^ ^]^ (listed in Table S9, Supporting Information), (Ti_1/3_Nb_1/3_Ta_1/3_)_2_FeC has a room‐temperature Curie point.

The field‐dependent magnetization curves of two magnetic MAX phases were clearly shown in Figure 3c,d. The maximum saturation magnetization of magnetic MAX phases has not been reached with an applied magnetic field increased to 10 000 Oe. A high saturation field is the hallmark of a magneto crystalline anisotropy that is caused by variations of fine‐grain arrangement.^[^ ^15^ ^]^ All the hysteresis loops showed a thin “*S*‐shape,” indicating the soft magnetic properties of the two samples.^[^ ^16^ ^]^ At 2 K, the magnetic hysteresis loops demonstrated low residual magnetization (*M*_r_), 5.21, and 3.50 emu g^−1^ for (Ti_1/3_Nb_1/3_Ta_1/3_)_2_FeC and (Ti_0.2_V_0.2_Nb_0.2_Ta_0.2_Zr_0.2_)_2_FeC, respectively. In addition, *M*_r_, *H*_c_ (coercive force), and *M*_s_ (maximum saturation magnetization) are gradually decrease with the temperature from 2 to 400 K (Tables S10 and S11, Supporting Information), which same as reported ternary magnetic MAX phases.^[^ ^17b^ ^]^ High saturation field and wide magnetic transition temperature range implied that there is no simple collinear ferromagnetic ordering of the two magnetic MAX phases.^[^ ^14a,b^ ^]^ The *S*‐shaped magnetic responses were illustrated throughout the temperature range of 5–200 K, and transforms into paramagnetic above 300 K.

As for Nb_2_FeC, the theoretical simulation indicates that the magnetism of Nb_2_FeC is mainly ascribed to the strong intralayer exchange interaction of the Fe layers. Similarly, in (Ti_1/3_Nb_1/3_Ta_1/3_)_2_FeC and (Ti_0.2_V_0.2_Nb_0.2_Ta_0.2_Zr_0.2_)_2_FeC, intralayer exchange interaction plays a very important role. However, there are multiprincipal elements (Ti, Nb, Ta) in (Ti_1/3_Nb_1/3_Ta_1/3_)_2_FeC. And the multiprincipal elements of M sites and the replacement of A layers lead lattice distortion and change lattice constants in MAX phases. This results in an enhanced coupling between the M‐A bonds of (Ti_1/3_Nb_1/3_Ta_1/3_)_2_FeC. As for why there is no room‐temperature ferromagnetic in (Ti_0.2_V_0.2_Nb_0.2_Ta_0.2_Zr_0.2_)_2_FeC, the possible reason is the increases of the chemical disorder of MAX phase, because of the introduction of more elements in M layers. It leads to a weak spin‐electron coupling, and reduces the magnetism of MAX phase. Thus, multiprincipal elements play an important role of tuning magnetic properties of MAX phases by altering the species and contents of elements.

### 2.3 EM Wave Absorption of Magnetic MAX Phases with Multiprincipal Elements

**Figure 4:**
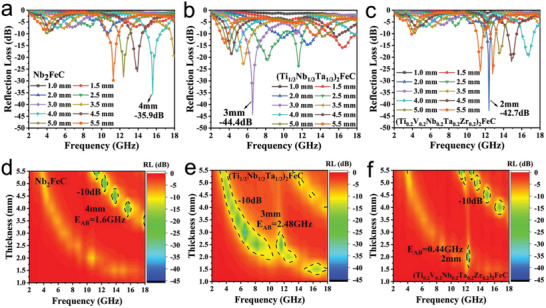
Reflection loss curves and its corresponding two‐dimensional contour mappings of: a,d) Nb_2_FeC; b,e) (Ti_1/3_Nb_1/3_Ta_1/3_)_2_FeC; and c,f) (Ti_0.2_V_0.2_Nb_0.2_Ta_0.2_Zr_0.2_)_2_FeC.

MAX phases as a candidate material for EM microwave absorption, there may be some new phenomena of magnetic MAX phase with multiprincipal elements for EM microwave absorbing performance. In general, the reflection loss (RL) value is used to evaluate the absorbing performance of the material. The EM wave absorption performance of the three samples was tested by a vector network analyzer. Generally, the smaller the RL value is, the stronger the absorbing performance is. **Figure** **4** is the RL curves and two‐dimensional contour mappings of Nb_2_FeC, (Ti_1/3_Nb_1/3_Ta_1/3_)_2_FeC, and (Ti_0.2_V_0.2_Nb_0.2_Ta_0.2_Zr_0.2_)_2_FeC with different thickness at 2–18 GHz, respectively. In Figure 4a, it was seen that when the thickness is 4 mm, the minimum RL value of Nb_2_FeC reaches −35.9 dB at 15.6 GHz, and the optimal bandwidth (E_AB_, RL< −10 dB) is 1.6 GHz (Figure 4d). In addition, as shown in Figure 4b, when the thickness is 3 mm, (Ti_1/3_Nb_1/3_Ta_1/3_)_2_FeC shows the strongest electromagnetic wave absorption with an RL value of −44.4 dB at 6.56 GHz, the effective bandwidth is 2.48 GHz (Figure 4e). Compared with Nb_2_FeC, (Ti_1/3_Nb_1/3_Ta_1/3_)_2_FeC has better absorption performance, lower matching thickness, and wider bandwidth width. In Figure 4c, under the condition of sample thickness of 2 mm and 12.4 GHz, the reflection loss value of (Ti_0.2_V_0.2_Nb_0.2_Ta_0.2_Zr_0.2_)_2_FeC reaches −42.7 dB, and the effective bandwidth is 0.44 GHz (Figure 4f). The best matching thickness of (Ti_0.2_V_0.2_Nb_0.2_Ta_0.2_Zr_0.2_)_2_FeC is smaller than that of other samples, and the minimum RL value of (Ti_0.2_V_0.2_Nb_0.2_Ta_0.2_Zr_0.2_)_2_FeC is not much different from that of (Ti_1/3_Nb_1/3_Ta_1/3_)_2_FeC. However, the effective bandwidth of (Ti_0.2_V_0.2_Nb_0.2_Ta_0.2_Zr_0.2_)_2_FeC is smaller than that of other samples. In short, with the increase of element composition of M‐sites and Fe atomic layers in MAX phases, the electromagnetic wave absorption performance of materials has been greatly improved.

**Figure 5:**
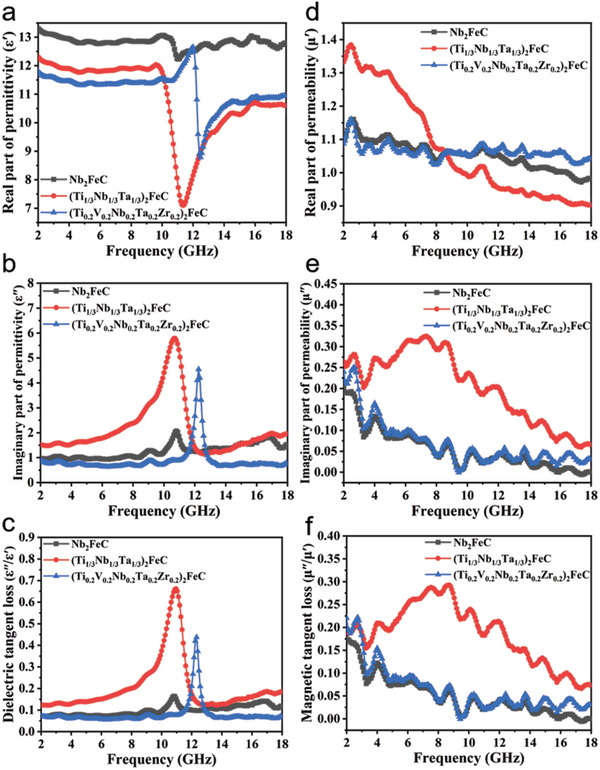
Electromagnetic parameters of Nb_2_FeC, (Ti_1/3_Nb_1/3_Ta_1/3_)_2_FeC, and (Ti_0.2_V_0.2_Nb_0.2_Ta_0.2_Zr_0.2_)_2_FeC: a) *ε*′, b) *ε*″, c) *ε*″/*ε*′, d) *µ*′, e) *µ*″, and f) *µ*″/*µ*′.

**Figure** **5** shows the electromagnetic parameters of Nb_2_FeC, (Ti_1/3_Nb_1/3_Ta_1/3_)_2_FeC, and (Ti_0.2_V_0.2_Nb_0.2_Ta_0.2_Zr_0.2_)_2_FeC. It is well known that the electromagnetic absorption properties of absorbing materials are closely related to the complex permittivity (*ε*_r_ = *ε*′‐ *jε″*) and complex permeability (*µ*_r_ = *µ′* − *jµ″*). Meanwhile, the tangent value of dielectric loss and magnetic loss (tan*δ*_*ε*_ = *ε*″/*ε*′ and tan*δ*_µ_ = *µ″/µ′*) are often used to estimate power loss.^[^ ^18^ ^]^

In Figure 5a, the *ε*′ value of Nb_2_FeC is higher than that of the other two samples. Notably, three samples exhibit concave peaks around 10–16 GHz, and (Ti_1/3_Nb_1/3_Ta_1/3_)_2_FeC has the greatest peak depression. As can be seen from Figure 5b, the *ε*″ value of (Ti_1/3_Nb_1/3_Ta_1/3_)_2_FeC is mostly higher than that of other samples, indicating that it has a strong dielectric loss capacity. Surprisingly, three samples have bulging peaks around 8–14 GHz, and the concave peak of (Ti_1/3_Nb_1/3_Ta_1/3_)_2_FeC is the most prominent. As discussed earlier about the lattice constants after the replacement reaction, it can be found that the *a*,*c* of (Ti_1/3_Nb_1/3_Ta_1/3_)_2_FeC changes the most. The bigger variation of the lattice constant of (Ti_1/3_Nb_1/3_Ta_1/3_)_2_FeC means that more polarization centers in (Ti_1/3_Nb_1/3_Ta_1/3_)_2_FeC than (Ti_0.2_V_0.2_Nb_0.2_Ta_0.2_Zr_0.2_)_2_FeC. Thus, the dielectric polarization of (Ti_1/3_Nb_1/3_Ta_1/3_)_2_FeC could be enhanced. Figure 5c shows the tangent value of the dielectric constant of the three samples, and the trend is the same as Figure 5b. Among them, (Ti_1/3_Nb_1/3_Ta_1/3_)_2_FeC has the highest *ε*″/*ε*′ value and its strongest dielectric loss capacity. In general, dielectric losses include polarization losses and conductivity losses. In addition, polarization losses include interface polarization and dipole polarization. Due to the increase of composition elements of MAX phases, the material is prone to polarization loss, which is conducive to improving the dielectric loss capacity. Notably, dielectric loss behavior can be further confirmed by the Debye relaxation theory.^[^ ^19^ ^]^

Since the three samples are magnetic, magnetic loss plays an important role in electromagnetic wave absorption, and the magnetic losses mainly include eddy current losses, natural resonances, and exchange resonances. Figure 5d shows the real part of the permeability (*µ*′) of the three samples. In the frequency range of 2–18 GHz, the *µ*′ value of (Ti_1/3_Nb_1/3_Ta_1/3_)_2_FeC continues to decrease (from 1.33 to 0.9), while *µ*′ value of Nb_2_FeC and (Ti_0.2_V_0.2_Nb_0.2_Ta_0.2_Zr_0.2_)_2_FeC remains unchanged. Surprisingly, the *µ*′ value of (Ti_1/3_Nb_1/3_Ta_1/3_)_2_FeC is the highest at 2–8.64 GHz, while it has the lowest *µ*′ value at 8.64–18 GHz. In Figure 5e, the *µ*″ value of (Ti_1/3_Nb_1/3_Ta_1/3_)_2_FeC is higher than that of other samples, indicating its strong magnetic loss ability. In addition, with the increase of frequency, the *µ*″ value of (Ti_1/3_Nb_1/3_Ta_1/3_)_2_FeC increases and then decreases from 4 to 12 GHz, while the *µ*″ values of the other two samples continue to decrease. The reason for this phenomenon is that there is nature magnetic resonance inside the (Ti_1/3_Nb_1/3_Ta_1/3_)_2_FeC. Figure 5f shows the tangent value of the permeability of the three samples, and the results show that the regularity is the same as Figure 5e, indicted (Ti_1/3_Nb_1/3_Ta_1/3_)_2_FeC has strong magnetic loss capability. Using the magnetic loss mechanism, electromagnetic waves are converted into heat energy and dissipated.^[^ ^20^ ^]^ In conclusion, MAX phases absorbing mechanism combined with dielectric loss and magnetic loss, and the dielectric loss is the main one, supplemented by magnetic loss. Compared with Nb_2_FeC, (Ti_1/3_Nb_1/3_Ta_1/3_)_2_FeC and (Ti_0.2_V_0.2_Nb_0.2_Ta_0.2_Zr_0.2_)_2_FeC have good absorption performance, it shows that the element control strategy (M and A‐sites) of MAX phase has an important influence on its wave absorption performance.

**Figure 6:**
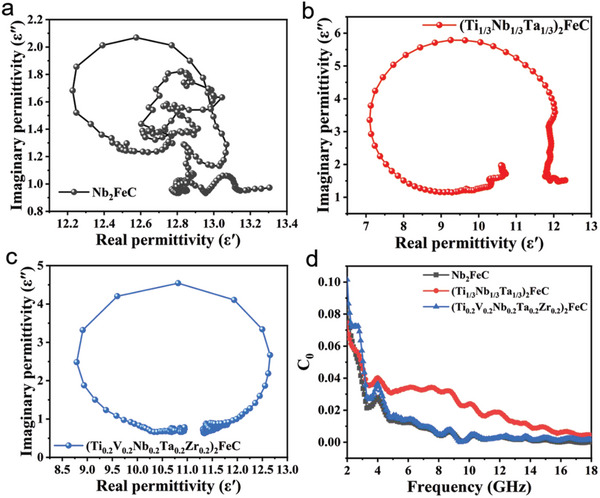
The Cole–Cole curve of: a) Nb_2_FeC, b) (Ti_1/3_Nb_1/3_Ta_1/3_)_2_FeC, and c) (Ti_0.2_V_0.2_Nb_0.2_Ta_0.2_Zr_0.2_)_2_FeC. d) The values *C*_0_ (*µ″* (*µ′*)^−2^ *f* ^−1^) of Nb_2_FeC, (Ti1/3Nb1/3Ta1/3)_2_FeC, and (Ti_0.2_V_0.2_Nb_0.2_Ta_0.2_Zr_0.2_)_2_FeC samples as a function of frequency.

As we all know, Debye relaxation is an important mechanism of EM wave absorption. The formula of Debye's theory is as follows:^[^ ^21^ ^]^

(1) $${\left(\varepsilon^{\prime }-\frac{\varepsilon_{s}+\varepsilon_{\infty }}{2}\right)}^{2}+{\left(\varepsilon^{\prime \prime }\right)}^{2}={\left(\frac{\varepsilon_{s}-\varepsilon_{\infty }}{2}\right)}^{2}$$

where *ε′* represents real part dielectric constant, *ε″* represents virtual part dielectric constant, *ε*_s_ represents static dielectric constant, and *ε*_∞_ represents high‐frequency dielectric constant. According to the Debye theory, we use the Cole–Cole semicircle to analyze the relaxation process of Nb_2_FeC, (Ti_1/3_Nb_1/3_Ta_1/3_)_2_FeC, and (Ti_0.2_V_0.2_Nb_0.2_Ta_0.2_Zr_0.2_)_2_FeC, respectively. In **Figure** **6**a, Nb_2_FeC has Cole–Cole semicircles with a small radius, indicating that its polarization loss ability is weak. However, (Ti_1/3_Nb_1/3_Ta_1/3_)_2_FeC and (Ti_0.2_V_0.2_Nb_0.2_Ta_0.2_Zr_0.2_)_2_FeC has a semicircle with a large radius (Figure 6b,c), indicating that the two samples have strong interfacial polarization and dipole polarization, which enhances the dielectric loss of the multiple‐element magnetic MXA phases. The addition of element with different radii causes lattice distortion, deforms the lattice interface, and is prone to interface polarization. In addition, the addition of more elements (in M‐sites) and isomorphous replacement (in A‐sites) with different radii causes more defects and vacancies in MAX phases, which forming defective dipoles. Under the alternating electric field, the pole distance of the dipole changes, forming a dipole polarization. Thus, there is a polarization peak in the range of 10–13 GHz. In short, the increase in the number of elements in M‐sites of MAX phases can improve its polarization loss, thereby improving the dielectric loss capacity, which is conducive to the absorption of electromagnetic waves. In addition, the resistivity of (Ti_0.2_V_0.2_Nb_0.2_Ta_0.2_Zr_0.2_)_2_FeC is greater than that of (Ti_1/3_Nb_1/3_Ta_1/3_)_2_FeC, which makes transportation of electrons more difficult. The magnetic losses of the three samples mainly include eddy current losses, natural resonances, and exchange resonances.^[^ ^22^ ^]^ The eddy current loss value is calculated by *C*_0_ = *µ*″ (*µ*′)^−2^ *f* ^−1^. When the *C*_0_ value is constant with the change of frequency, it indicates that there is eddy current loss in the material. When the value of *C*_0_ changes, it indicates that there is magnetic resonance phenomenon in the material.^[^ ^23^ ^]^ As shown in Figure 6d, the *C*_0_ value of (Ti_1/3_Nb_1/3_Ta_1/3_)_2_FeC gradually decreases within 2–18 GHz, indicating that its magnetic loss exists both eddy current loss and magnetic resonance phenomenon. However, for Nb_2_FeC and (Ti_0.2_V_0.2_Nb_0.2_Ta_0.2_Zr_0.2_)_2_FeC, the *C*_0_ value of these two samples decreases significantly at 2–10 GHz, indicating the existence of magnetic resonance phenomenon in these two samples at 2–10 GHz. Then, the *C*_0_ values of these two samples remain unchanged from 10 to 18 GHz, indicating that there is only eddy current loss.^[^ ^24^ ^]^

**Figure 7:**
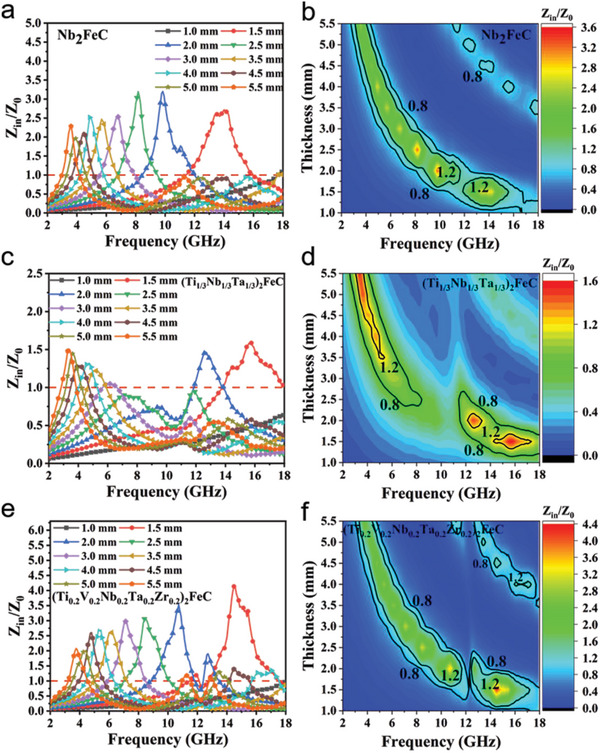
Impedance match (*Z*_in_/*Z*_0_) curves and its corresponding two‐dimensional contour mappings of: a,b) Nb_2_FeC; c,d) (Ti_1/3_Nb_1/3_Ta_1/3_)_2_FeC; and e,f) (Ti_0.2_V_0.2_Nb_0.2_Ta_0.2_Zr_0.2_)_2_FeC.

In addition, impedance matching is also one of the important factors affecting absorption performance. When the value of *Z*_in_ */Z*_0_ is close to 1, the impedance matching is good, as shown in **Figure** **7**. The *Z*_in_ */Z*_0_ value of (Ti_1/3_Nb_1/3_Ta_1/3_)_2_FeC has a large area in the range of 0.8–1.2 (Figure 7d), indicating that it has a good impedance matching performance. Moreover, when the thickness is 3 mm, its *Z*_in_ */Z*_0_ value is closest to 1 (Figure 7c). The optimum matching thickness of (Ti_1/3_Nb_1/3_Ta_1/3_)_2_FeC is 3 mm. In addition, in Figure 7e,f, the *Z*_in_ */Z*_0_ value of (Ti_0.2_V_0.2_Nb_0.2_Ta_0.2_Zr_0.2_)_2_FeC in the range of 0.8–1.2 is smaller than that of (Ti_1/3_Nb_1/3_Ta_1/3_)_2_FeC, indicating (Ti_0.2_V_0.2_Nb_0.2_Ta_0.2_Zr_0.2_)_2_FeC impedance matching performance is poor. In addition, when the thickness is 2 mm, its *Z*_in_ */Z*_0_ value close to 1 area is not obvious, therefore (Ti_0.2_V_0.2_Nb_0.2_Ta_0.2_Zr_0.2_)_2_FeC has a small bandwidth (Figure 4c). For Nb_2_FeC (Figure 7a,b), its *Z*_in_ */Z*_0_ value in the range of 0.8–1.2 is similar to (Ti_0.2_V_0.2_Nb_0.2_Ta_0.2_Zr_0.2_)_2_FeC, indicated the impedance matching performance is poor. When the thickness is 4 mm, the *Z*_in_ */Z*_0_ value is closest to 1, so the best matching thickness of Nb_2_FeC is 4 mm. These results indicate that (Ti_1/3_Nb_1/3_Ta_1/3_)_2_FeC and (Ti_0.2_V_0.2_Nb_0.2_Ta_0.2_Zr_0.2_)_2_FeC are expected to be widely used in the field of electromagnetic wave absorption, with the advantages of excellent microwave absorbing performance, low matching thickness, and wide frequency band.

## 3 Conclusion

In summary, new (Ti_1/3_Nb_1/3_Ta_1/3_)_2_FeC and (Ti_0.2_V_0.2_Nb_0.2_Ta_0.2_Zr_0.2_)_2_FeC magnetic MAX phases have been successfully synthesized by the isomorphous replacement reaction with Lewis acid salt. The MAX phases are exhibited intrinsic ferromagnetic properties, and the Curie temperature of (Ti_1/3_Nb_1/3_Ta_1/3_)2FeC is 302 K. It demonstrates that multiprincipal elements in MAX phases are offered a route to tune magnetic behavior. Meanwhile, the EM wave absorption properties of the two magnetic MAX phases were investigated. The results show that (Ti_1/3_Nb_1/3_Ta_1/3_)_2_FeC possesses enhanced EM absorption performance, compared with Nb_2_FeC and (Ti_0.2_V_0.2_Nb_0.2_Ta_0.2_Zr_0.2_)_2_FeC. Specifically, the minimum RL value of (Ti_1/3_Nb_1/3_Ta_1/3_)_2_FeC is −44.4 dB at 6.56 GHz with a thickness of 3 mm, and the effective bandwidth is 2.48 GHz. Hence, it is important that magnetic MAX phases with multiprincipal elements are expected to be potential EM absorbers with the combination of electrical loss and magnetic loss.

## 4 Experimental Section

### Synthesis Details

High‐purity commercial powders of titanium (99.0 wt%, 1 µm), vanadium (98 wt%, 10–20 µm), niobium (99 wt%, 45 µm), zirconium hydride (99 wt%, 30 µm), tantalum (99 wt%, 25 µm), and aluminum (99 wt%, 1 µm) were all bought from Shanghai PanTian nano Materials Co. Ltd. Graphite (99.9 wt%, 5000 mesh), sodium chloride (NaCl, 98 wt%), potassium chloride (KCl, 98 wt%), lithium chloride (LiCl, 98 wt%), ferrous chloride (FeCl_2_, 99.5 wt%), and hydrochloric acid (HCl, 98 wt%) were obtained from Aladdin Chemical Reagent, China.

The starting powders were mixed in an agate mortar with a stoichiometric ratio of M:Al:C = 2:1.1:1 (aluminum had a low melting point, thus added more Al power to prevent loss in the reaction), and elements in M‐site were equimolal. Additionally, the starting powders were mixed with ethanol, grinded for 30 min, and dried in vacuum for 12 h. Then, the mixed powders were filled in a graphite die and heated to 1300 °C for 30 min with a heating rate of 50 °C min^−1^ in Ar by spark plasma sintering (SPS). No pressure was applied during the whole reaction process. After the end of reaction, the as‐prepared powders were washed in 3 m HCl for 48 h, filtered, and dried at 40 °C for 3 h in vacuum. Finally, (Ti_1/3_Nb_1/3_Ta_1/3_)_2_AlC and (Ti_0.2_V_0.2_Nb_0.2_Ta_0.2_Zr_0.2_)_2_AlC MAX phases were obtained.

The magnetic MAX phases were obtained through isomorphous replacement reaction of multiprincipal (Ti_1/3_Nb_1/3_Ta_1/3_)_2_AlC and (Ti_0.2_V_0.2_Nb_0.2_Ta_0.2_Zr_0.2_)_2_AlC MAX phases with Lewis acid salts (FeCl_2_).^[^ ^17^ ^]^ The MAX phases precursor of (Ti_1/3_Nb_1/3_Ta_1/3_)_2_AlC and (Ti_0.2_V_0.2_Nb_0.2_Ta_0.2_Zr_0.2_)_2_AlC and FeCl_2_ was mixed with a molar ratio of 1:1.5. The prepared mixture was loaded into alumina crucible, and put into tube furnace. The reaction process was at 650 °C for 7 h under the protection of Ar atmosphere. After the end of the reaction, the product was washed in 3 m HCl solution for 20 h to remove the by‐product of Fe. Then, the target product (Ti_1/3_Nb_1/3_Ta_1/3_)_2_FeC and (Ti_0.2_V_0.2_Nb_0.2_Ta_0.2_Zr_0.2_)_2_FeC MAX phases were obtained.

### Materials Characterization

The as‐prepared powders were analyzed by X‐ray diffraction (XRD, D8 Advance, Bruker AXS, Germany) with Cu K_*α*_ radiation. X‐ray diffractograms were collected at a step size of 0.02° 2*θ* with a collection time of 1 s per step. The Rietveld refinement of powder XRD patterns of MAX phases were by Total Pattern Solution (*TOPAS‐Academic v6*). The microstructure and chemical composition were examined by a scanning electron microscopy (SEM, QUANTA 250 FEG, FEI, USA) and a transmission electron microscope (TEM; Spectra 300, USA) equipped with an energy‐dispersive spectrometer (EDS). The samples analyzed in TEM were cut and thinned using a dual‐beam scanning electron microscope‐focused ion beam (FIB; Thermo Scientific Helios‐G4‐CX, USA). The magnetic properties were detected by DC‐Superconducting‐Quantum‐Interface‐Devices (SQUID, 7 T).

### EM Wave Absorption Performance Test

The complex permittivity and permeability of the three samples were measured by a vector network analyzer in the range of 2–18 GHz using the coaxial method. In this work, three different samples were mixed with paraffin at a mass ratio of 8:2 and evenly pressed into a ring with an inner diameter of 3.04 mm and an outer diameter of 7.0 mm, respectively. Usually, the reflection loss value was used to express the microwave absorption performance. According to the transmission line theory, the corresponding formula is as follows:^[^ ^25^ ^]^

(2) $$\mathrm{RL}(\mathrm{dB})=20\log\left|\frac{Z_{\mathrm{in}}-Z_{0}}{Z_{\mathrm{in}}+Z_{0}}\right|$$

(3) $$Z_{\mathrm{in}}=Z_{0}\sqrt{\frac{\mu_{r}}{\varepsilon_{r}}}\tanh\left[j\left(\frac{2\pi }{c}\right)fd\sqrt{\mu_{r}\varepsilon_{r}}\right]$$

where *Z*_in_, *Z*_0_, *c*, *f*, and *d* denote the normalized input impendence, the impedance of air, the light speed in a vacuum, the frequency of incident EM waves, and the thickness of absorbers, respectively.

## Conflict of Interest

The authors declare no conflict of interest.

## Supporting information

Supporting Information

## Data Availability

The data that support the findings of this study are available from the corresponding author upon reasonable request.

## References

[1] a) M. Green , X. Chen , J. Materiomics 2019, 5, 503;

[2] a) M. Sokol , V. Natu , S. Kota , M. W. Barsoum , Trends Chem. 2019, 1, 210;

[3] a) M. Barsoum , Encyclopedia of Materials: Science and Technology, Elsevier, Amsterdam, Netherlands, 2006, 1;

[4] Y. Liu , F. Luo , W. Zhou , D. Zhu , J. Alloys Compd. 2013, 576, 43.

[5] Y. Shi , F. Luo , Y. Liu , W. Zhou , X. Zhang , Int. J. Appl. Ceram. Technol. 2015, 12, E172.

[6] Y. Zhang , J. Wen , L. Zhang , H. Lu , Y. Guo , X. Ma , M. Zhang , J. Yin , L. Dai , X. Jian , L. Yin , J. Xie , D. Liang , L. Deng , J. Alloys Compd. 2021, 860, 157896.

[7] a) J. He , L. Deng , H. Luo , L. He , D. Shan , S. Yan , S. Huang , J. Magn. Magn. Mater. 2019, 473, 184;

[8] a) S. Zhao , L. Chen , H. Xiao , J. Huang , Y. Li , Y. Qian , T. Zheng , Y. Li , L. Cao , H. Zhang , H. Liu , Y. Wang , Q. Huang , C. Wang , Acta Mater. 2022, 238, 118222;

[9] W. Luo , Y. Liu , C. Wang , D. Zhao , X. Yuan , L. Wang , J. Zhu , S. Guo , X. Kong , J. Mater. Chem. C 2021, 9, 7697.

[10] L. Qiao , J. Bi , G. Liang , C. Liu , Z. Yin , Y. Yang , H. Wang , S. Wang , M. Shang , W. Wang , J. Mater. Sci. Technol. 2023, 137, 112.

[11] B. Zhao , Y. Li , Q. Zeng , L. Wang , J. Ding , R. Zhang , R. Che , Small 2020, 16, 2003502. 10.1002/smll.202003502 32893495

[12] a) J. Kroder , J. Gooth , W. Schnelle , G. H. Fecher , C. Felser , 2019, 9, 055327;

[13] P. M. Morse , Mater. Res. Lett. 1932, 76, 326.

[14] a) A. Ingason , A. Petruhins , M. Dahlqvist , F. Magnus , A. Mockute , B. Alling , L. Hultman , I. A. Abrikosov , P. Persson , J. Rosén , Mater. Res. Lett. 2014, 2, 89;

[15] R. Meshkian , A. S. Ingason , U. B. Arnalds , F. Magnus , J. Lu , J. Rosen , APL. Mater 2015, 3, 076102.

[16] a) A. S. Ingason , A. Mockute , M. Dahlqvist , F. Magnus , S. Olafsson , U. B. Arnalds , B. Alling , I. A. Abrikosov , B. Hjörvarsson , P. O. Å. Persson , J. Rosen , Phys. Rev. Lett. 2013, 110, 195502;. 23705717 10.1103/PhysRevLett.110.195502

[17] a) M. Li , J. Lu , K. Luo , Y. Li , K. Chang , K. Chen , J. Zhou , J. Rosen , L. Hultman , P. Eklund , P. O. A. Persson , S. Du , Z. Chai , Z. Huang , Q. Huang , J. Am. Chem. Soc. 2019, 141, 4730;. 30821963 10.1021/jacs.9b00574

[18] a) B. Zhao , Y. Li , H. Ji , P. Bai , S. Wang , B. Fan , X. Guo , R. Zhang , Carbon 2021, 176, 411;

[19] K. Iwauchi , Jpn. J. Appl. Phys. 1971, 10, 1520.

[20] T. Chen , F. Deng , J. Zhu , C. Chen , G. Sun , S. Ma , X. Yang , J. Mater. Chem. 2012, 22, 15190.

[21] a) K. Zhang , M. Ye , A. Han , J. Yang , J. Solid State Chem. 2019, 277, 68;

[22] Q. Liu , Q. Cao , H. Bi , C. Liang , K. Yuan , W. She , Y. Yang , R. Che , Adv. Mater. 2016, 28, 486. 26588359 10.1002/adma.201503149

[23] L. Liang , R. Yang , G. Han , Y. Feng , B. Zhao , R. Zhang , Y. Wang , C. Liu , ACS Appl. Mater. Interfaces 2020, 12, 2644. 31854182 10.1021/acsami.9b18504

[24] L. Liang , Q. Li , X. Yan , Y. Feng , Y. Wang , H.‐B. Zhang , X. Zhou , C. Liu , C. Shen , X. Xie , ACS Nano 2021, 15, 6622. 33780231 10.1021/acsnano.0c09982

[25] a) Y. Li , Y. Qing , Y. Zhou , B. Zhao , Q. Zhi , B. Fan , R. Zhang , Compos. B Eng. 2021, 213, 108731;

