# Supplementary material for: Multiprincipal Element M2FeC (M = Ti,V,Nb,Ta,Zr) MAX Phases with Synergistic Effect of Dielectric and Magnetic Loss
Source: Adv Sci (Weinh). 2023 Feb 2;10(10):2206877. doi: 10.1002/advs.202206877 (PMC10074122; doi:10.1002/advs.202206877)
Supplement: Supplementary file 1 — Supporting Information [file ADVS-10-2206877-s001.pdf]

ADVANCED SCIENCE
Open Access

Supporting Information

for *Adv. Sci.*, DOI 10.1002/advs.202206877

Multiprincipal Element M_2FeC (M = Ti,V,Nb,Ta,Zr) MAX Phases with Synergistic Effect of Dielectric and Magnetic Loss

Lu Chen, Youbing Li, Biao Zhao, Shanshan Liu, Huibin Zhang, Ke Chen, Mian Li, Shiyu Du, Faxian Xiu, Renchao Che*, Zhifang Chai and Qing Huang**

Support Information

Multi-principal element M_2FeC (M=Ti,V,Nb,Ta,Zr) MAX phases with synergistic effect of dielectric and magnetic loss

Lu Chen[1,2,3#], *Youbing Li*[1,3#], *Biao Zhao*[4*], *Shanshan Liu*[5], *Huibin Zhang*[4], *Ke Chen*[1,3], *Mian Li*[1,3], *Shiyu Du*[1,3], *Faxian Xiu*[5], *Renchao Che*[4*], *Zhifang Chai*[1,3], *Qing Huang*[1,3*]

[1]Engineering Laboratory of Advanced Energy Materials, Ningbo Institute of Materials Technology and Engineering, Chinese Academy of Sciences, Ningbo, Zhejiang 315201, China.

[2]University of Chinese Academy of Sciences, 19 A Yuquan Rd, Shijingshan District, Beijing 100049, China.

[3]Qianwan Institute of CNiTECH, Ningbo, 315336, China

[4]Laboratory of Advanced Materials, Shanghai Key Lab of Molecular Catalysis and Innovative Materials, School of Microelectronics, Fudan University, Shanghai 200438, China

[5]State Key Laboratory of Surface Physics and Department of Physics, Fudan University, Shanghai 200433, China

#Lu Chen and Youbing Li contributed equally to this work.

*To whom correspondence may be addressed. Email:

zhao_biao@fudan.edu.cn, rcche@fudan.edu.cn, huangqing@nimte.ac.cn

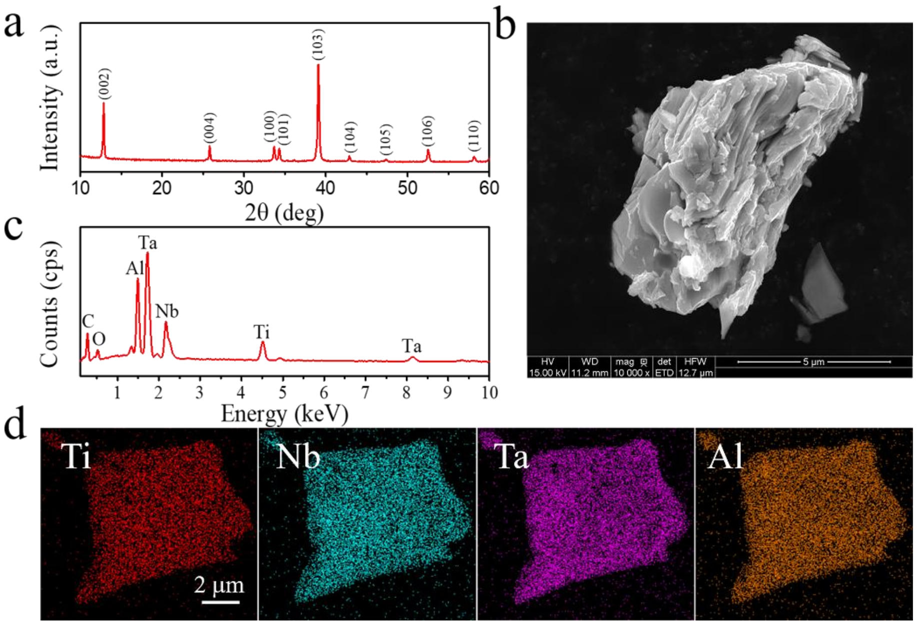

Fig. S1. (a) XRD pattern of the $(Ti_{1/3}Nb_{1/3}Ta_{1/3})_2AlC$ powder after acid treatment. (b) SEM image of the $(Ti_{1/3}Nb_{1/3}Ta_{1/3})_2AlC$ powder. (c) Corresponding energy-dispersive spectroscopy (EDS) spectrum. (d) EDS mapping of Ti-K_α, Nb-L_α, Ta-L_α and Al-K_α signals.

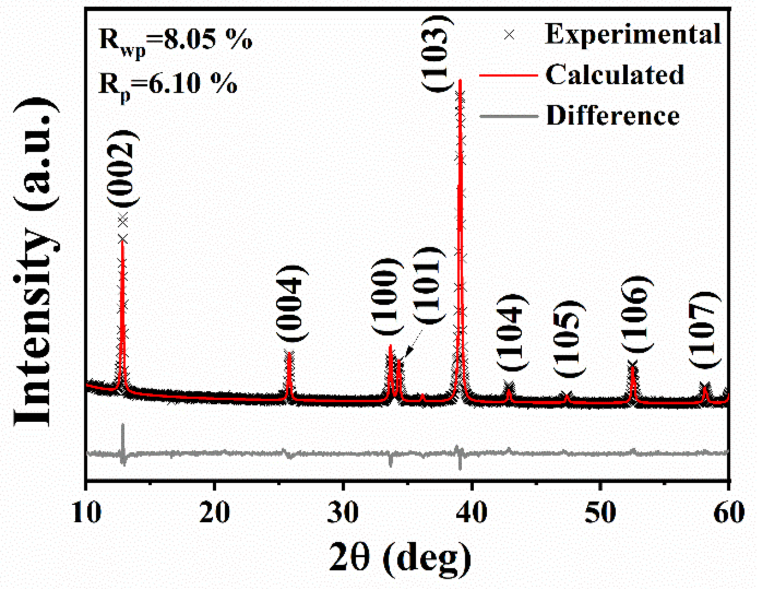

Fig. S2. XRD Rietveld refinements of $(Ti_{1/3}Nb_{1/3}Ta_{1/3})_2AlC$.

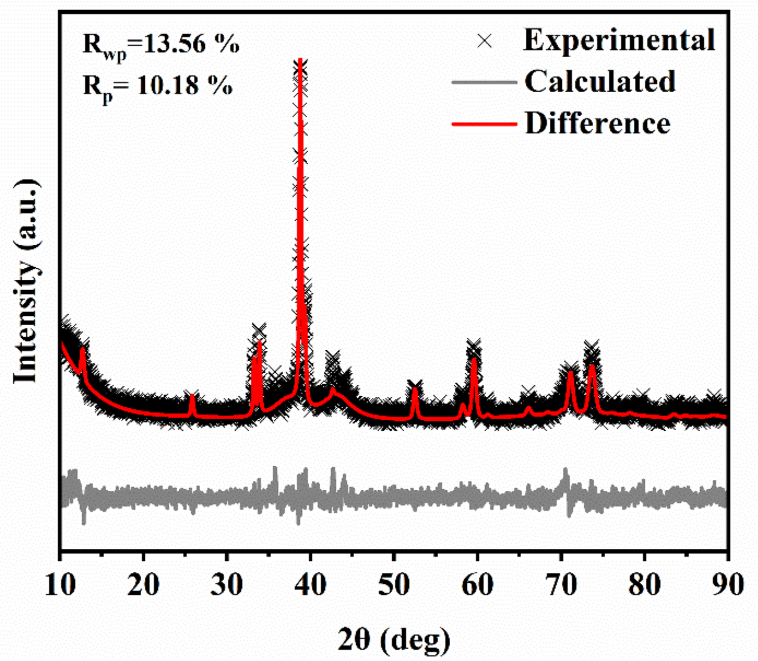

Fig. S3. XRD Rietveld refinements of $(Ti_{1/3}Nb_{1/3}Ta_{1/3})_2FeC$.

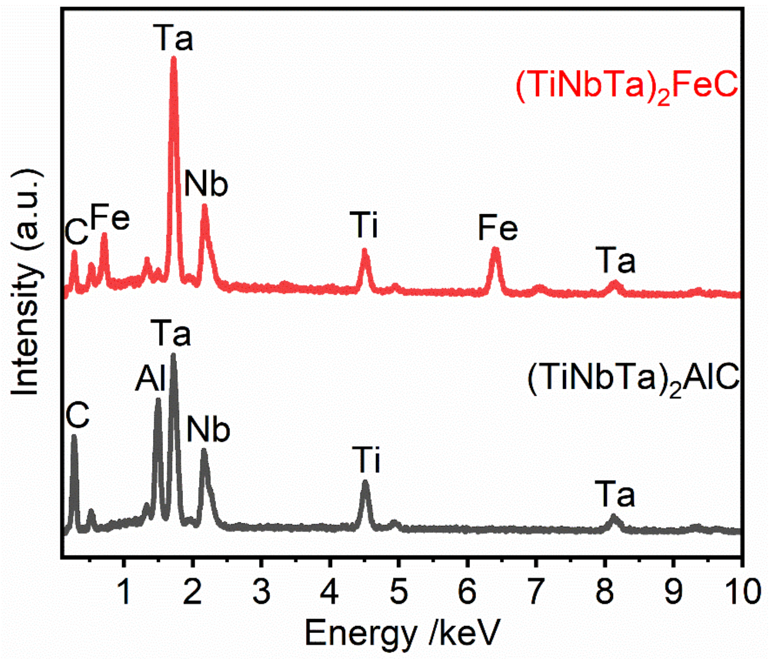

Fig. S4. Energy-dispersive spectroscopy (EDS) spectrum of $(Ti_{1/3}Nb_{1/3}Ta_{1/3})_2AlC$ and $(Ti_{1/3}Nb_{1/3}Ta_{1/3})_2FeC$.

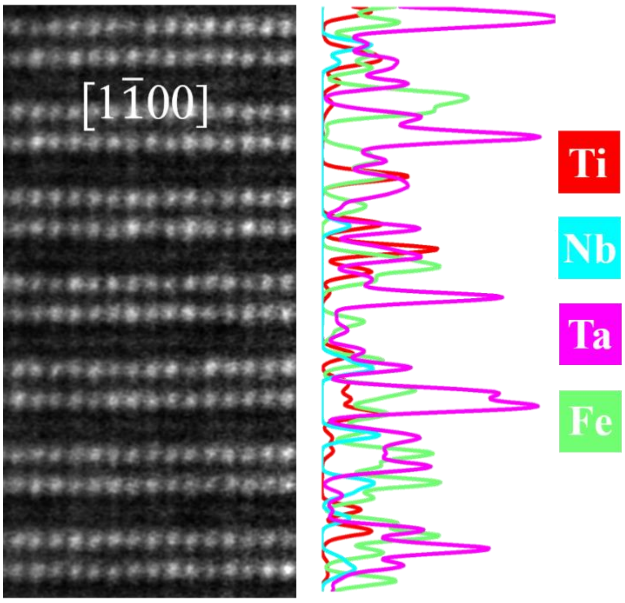

Fig. S5. lattice-resolved EDS line profile of $(Ti_{1/3}Nb_{1/3}Ta_{1/3})_2FeC$

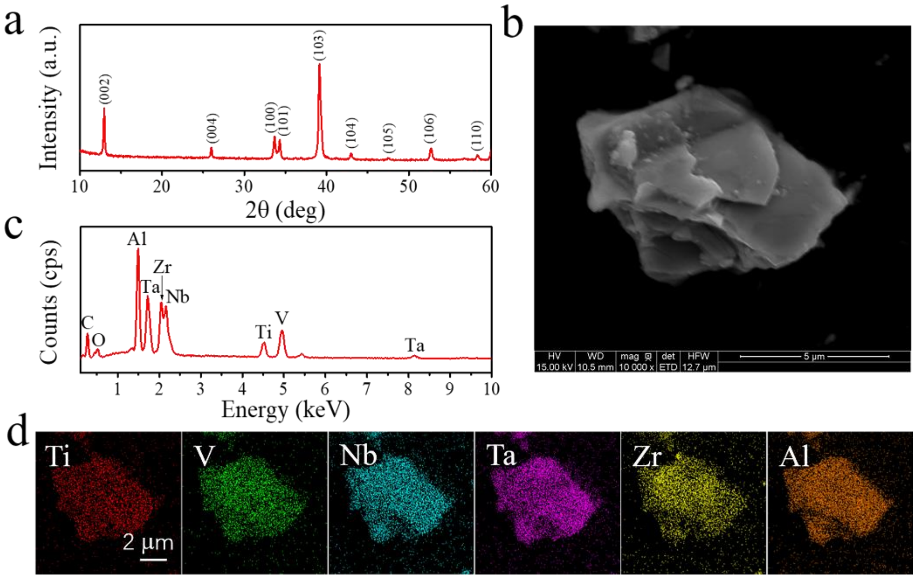

Fig. S6. (a) XRD pattern of the $(Ti_{0.2}V_{0.2}Nb_{0.2}Ta_{0.2}Zr_{0.2})_2AlC$ powder after acid treatment. (b) SEM image of the $(Ti_{0.2}V_{0.2}Nb_{0.2}Ta_{0.2}Zr_{0.2})_2AlC$ powder. (c) Corresponding energy-dispersive spectroscopy (EDS) spectrum. (d) EDS mapping of Ti-K_α, V- K_α, Nb-L_α, Ta-L_α, Zr- L_α and Al-K_α signals.

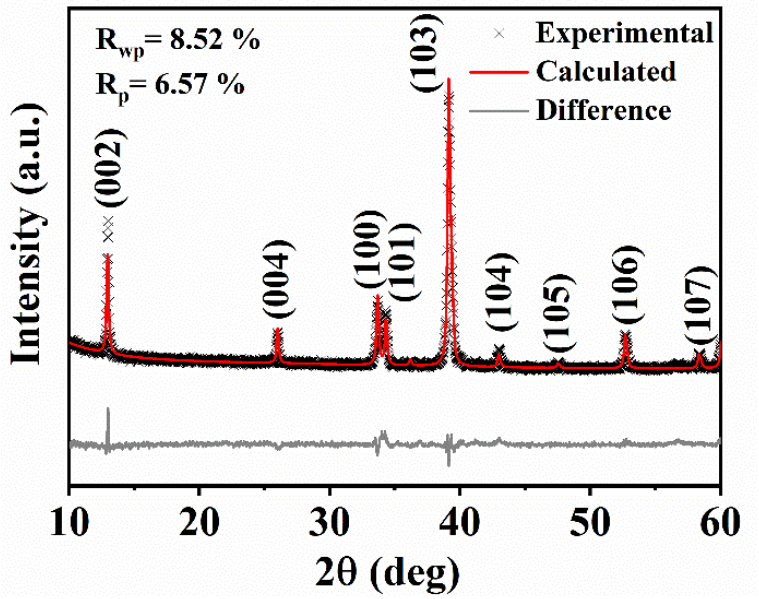

Fig. S7. XRD Rietveld refinements of $(Ti_{0.2}V_{0.2}Nb_{0.2}Ta_{0.2}Zr_{0.2})_2AlC$.

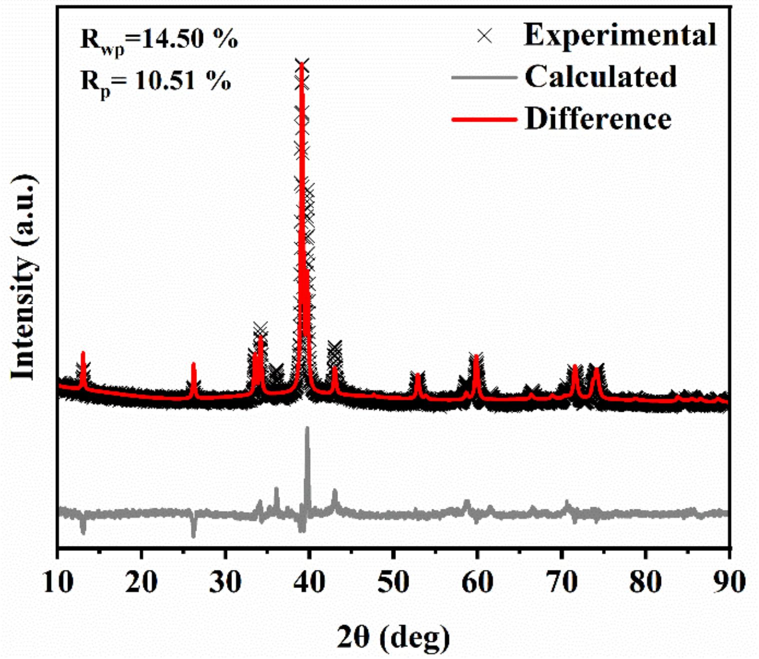

Fig. S8. XRD Rietveld refinements of $(Ti_{0.2}V_{0.2}Nb_{0.2}Ta_{0.2}Zr_{0.2})_2FeC$.

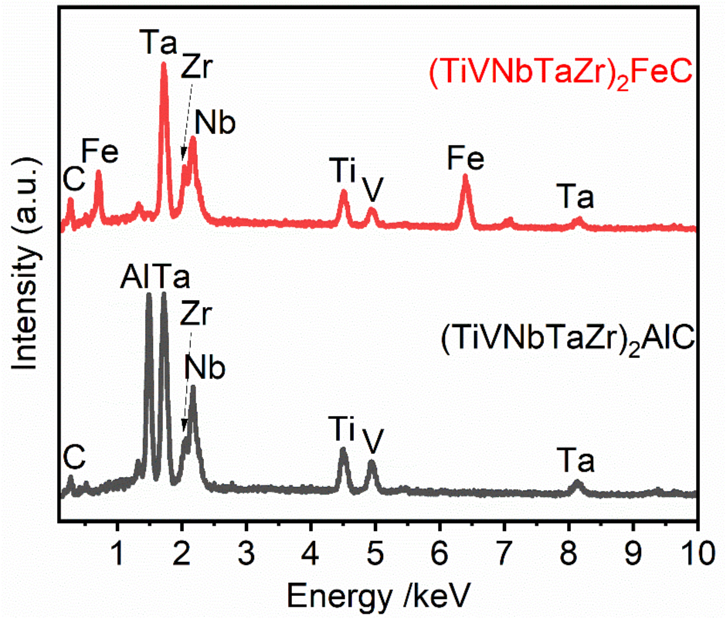

Fig. S9. Energy-dispersive spectroscopy (EDS) spectrum of $(Ti_{0.2}V_{0.2}Nb_{0.2}Ta_{0.2}Zr_{0.2})_2AlC$ and $(Ti_{0.2}V_{0.2}Nb_{0.2}Ta_{0.2}Zr_{0.2})_2FeC$.

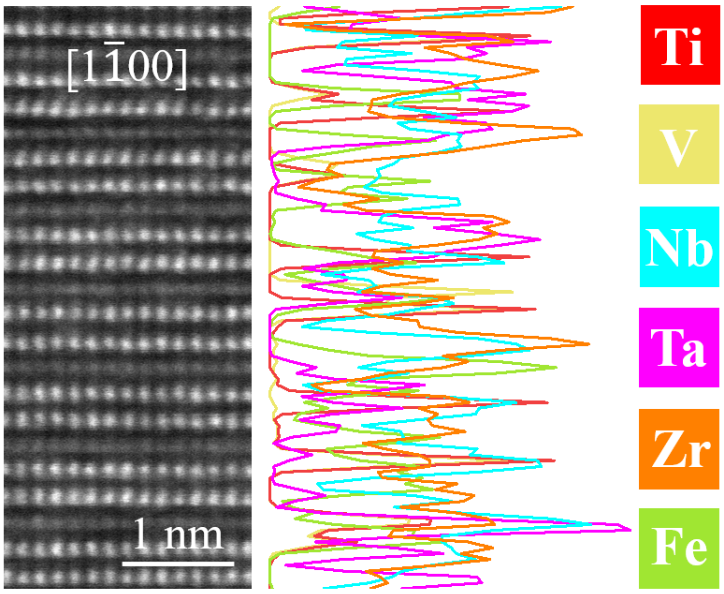

Fig. S10. lattice-resolved EDS line profile of $(Ti_{0.2}V_{0.2}Nb_{0.2}Ta_{0.2}Zr_{0.2})_2FeC$

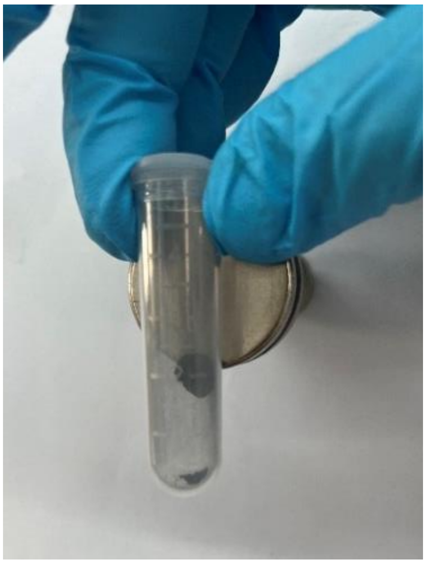

Fig. S11. The $(Ti_{1/3}Nb_{1/3}Ta_{1/3})_2)_2FeC$ powder hangs on the wall when it is close to the magnet.

Table S1. Diffraction peaks of $(Ti_{1/3}Nb_{1/3}Ta_{1/3})_2AlC$ and their positions

2θ (°)	*d* (Å)	*I/I1*(%)	FWHM	*hkl*
12.841	6.8883	57.5	0.164	002
25.794	3.4511	15.2	0.189	004
33.672	2.6595	15.1	0.234	100
34.322	2.6106	13.0	0.226	101
39.082	2.3029	100.0	0.242	103
42.855	2.1085	5.8	0.217	104
47.355	1.9181	2.4	0.306	105
52.473	1.7424	12.6	0.265	106
58.098	1.5864	5.3	0.287	107
60.152	1.5370	9.3	0.308	110

Table S2 Diffraction peaks of $(Ti_{1/3}Nb_{1/3}Ta_{1/3})_2FeC$ and their positions

2θ (°)	*d* (Å)	*I/I1*(%)	FWHM	*hkl*
12.691	6.9692	7.3	0.102	002
25.791	3.4515	5.7	0.245	004
33.266	2.6911	19.9	0.214	100
33.838	2.6468	24.3	0.240	101
35.743	2.5100	10.4	0.243	102
38.798	2.3191	100.0	0.334	103
39.286	2.2914	33.0	0.457	006
42.708	2.1154	12.6	0.235	104
52.619	1.7379	8.9	0.372	106
58.281	1.5818	6.1	0.297	107
59.534	1.5515	20.4	0.387	110
61.110	1.5152	5.5	0.279	112

Table S3. The atomic percentage of $(Ti_{1/3}Nb_{1/3}Ta_{1/3})_2AlC$ and $(Ti_{1/3}Nb_{1/3}Ta_{1/3})_2FeC$

sample	Element (at. %)					
	Ti	Nb	Ta	Al	Fe	C
$(Ti_{1/3}Nb_{1/3}Ta_{1/3})_2AlC$	7.97	8.50	7.90	11.54	-	64.09
$(Ti_{1/3}Nb_{1/3}Ta_{1/3})_2FeC$	8.0	6.3	7.5	0.3	18.5	45.7

Table S4 Atomic positions and composition of $(Ti_{1/3}Nb_{1/3}Ta_{1/3})_2FeC$

Element	x	y	z	Occ
Ti	1/3	2/3	0.5918	0.332
Nb	1/3	2/3	0.5914	0.334
Ta	1/3	2/3	0.5914	0.334
Fe	1/3	2/3	0.25	1
C	0	0	0	1

Table S5. Diffraction peaks of $(Ti_{0.2}V_{0.2}Nb_{0.2}Ta_{0.2}Zr_{0.2})_2AlC$ and their positions

2θ (º)	*d* (Å)	*I*/*I1*(%)	FWHM	*hkl*
12.976	6.8170	50.0	0.169	002
26.009	3.4231	11.3	0.203	004
33.691	2.6581	23.6	0.303	100
34.303	2.6120	20.0	0.300	101
39.141	2.2996	100.0	0.307	103
42.954	2.1039	7.0	0.294	104
47.554	1.9105	2.2	0.256	105
52.706	1.7353	12.8	0.318	106
58.374	1.5795	5.4	0.282	107
60.111	1.5380	12.6	0.332	110

Table S6. Diffraction peaks of $(Ti_{0.2}V_{0.2}Nb_{0.2}Ta_{0.2}Zr_{0.2})_2FeC$ and their positions

2θ (º)	*d* (Å)	*I*/*I1*(%)	FWHM	*hkl*
13.079	6.7635	4.1	0.122	002
26.281	3.3882	2.4	0.205	004
33.540	2.6696	16.9	0.335	100
34.184	2.6208	21.9	0.328	101
36.077	2.4876	8.7	0.208	102
39.129	2.3003	100.0	0.280	103
39.753	2.2656	30.4	0.244	006
43.023	2.1007	15.3	0.314	104
52.893	1.7296	7.0	0.321	106
58.715	1.5712	4.6	0.331	107
59.864	1.5437	15.0	0.356	110
61.538	1.5057	3.7	0.225	112

Table S7 Atomic positions and composition of $(Ti_{0.2}V_{0.2}Nb_{0.2}Ta_{0.2}Zr_{0.2})_2FeC$

Element	x	y	z	Occ
Ti	1/3	2/3	0.5910	0.2
V	1/3	2/3	0.5928	0.2
Nb	1/3	2/3	0.5967	0.2
Ta	1/3	2/3	0.5901	0.2
Zr	1/3	2/3	0.6007	0.2
Fe	1/3	2/3	0.25	1
C	0	0	0	1

Table S8. The atomic percentage of $(Ti_{0.2}V_{0.2}Nb_{0.2}Ta_{0.2}Zr_{0.2})_2AlC$ and $(Ti_{0.2}V_{0.2}Nb_{0.2}Ta_{0.2}Zr_{0.2})_2FeC$

sample	Element (at. %)							
	Ti	V	Nb	Ta	Zr	Al	Fe	C
$(TiVNbTaZr)_2AlC$	6.82	6.22	7.55	7.85	4.51	14.59	-	52.46
$(TiVNbTaZr)_2FeC$	9.1	4.2	8.0	7.9	4.4	0.2	24.4	37.7

Table S9. The magnetic properties compared with other MAX phases

MAX phases	T_c (K)	Ref.
$(Ti_{1/3}Nb_{1/3}Ta_{1/3})_2FeC$	302	this work
$(Ti_{0.2}V_{0.2}Nb_{0.2}Ta_{0.2}Zr_{0.2})_2FeC$	235	this work
Ta_2FeC	291	Li *et. al*
Ti_2FeN	208	Li *et. al*
Nb_2FeC	281	Li *et. al*
Mn_2GaC	<230	Ingason *et. al*
$(Cr_{0.85}Mn_{0.15})_2GeC$	205	Liu *et. al*
$(Cr_{1.95}Fe_{0.05})_2GeC$	250	Lin *et. al*
$(CrFe)_2AlC$	40	Hamm *et. al*
$(Cr_{0.5}Mn_{0.5})_2AuC$	100	Lai *et. al*

Table S10. The coercive force (H_c), residual magnetization (M_r) and maximum saturation magnetization (M_s) of the $(Ti_{1/3}Nb_{1/3}Ta_{1/3})_2FeC$ at different temperature under ZFC in the magnetic field range of -1T to 1T.

T (K)	H_c (Oe)	M_r (emu/g)	M_s (emu/g)
2	320.06	5.21	19.22
50	160.05	3.17	19.09
100	120.08	2.40	16.77
200	39.80	0.82	10.27
250	0.17	0.14	6.38
300	0.03	0.02	3.11
350	0	0.01	0.98

Table S11. The coercive force (H_c), residual magnetization (M_r) and maximum saturation magnetization (M_s) of the $(Ti_{0.2}V_{0.2}Nb_{0.2}Ta_{0.2}Zr_{0.2})_2FeC$ at different temperature under ZFC in the magnetic field range of -1T to 1T.

T (K)	H_c (Oe)	M_r (emu/g)	M_s (emu/g)
2	120.02	3.50	25.36
50	39.88	1.69	23.51
100	39.98	0.90	20.70
200	0.04	0.36	11.45
250	0.12	0.02	5.49
300	0.11	0.03	2.95
350	0.06	0.02	1.88
